# Stéatose hépatique non alcoolique : diagnostic et traitement en 2022

**DOI:** 10.1007/s43472-023-00091-9

**Published:** 2023-03-07

**Authors:** Nikoletta Maria Tagkou, Nicolas Goossens

**Affiliations:** Service de Gastroentérologie et d’Hépatologie, Hôpitaux Universitaires de Genève (HUG), Rue Gabrielle-Perret-Gentil 4, 1211 Genève 14, Suisse

**Keywords:** Maladie du foie, Obésité, Diabète de type 2, Fibrose du foie, Cirrhose, Lebererkrankung, Fettleibigkeit, Typ-2-Diabetes, Leberfibrose, Zirrhose, malattie del fegato, obesità, diabete di tipo 2, fibrosi epatica, cirrosi

## Abstract

La NAFLD (*Non Alcoholic Fatty Liver Disease*) est la manifestation hépatique d’un trouble métabolique multisystémique. Elle est la principale cause de maladie hépatique au niveau mondial, avec une prévalence croissante. Bien qu’il s’agisse principalement d’une maladie silencieuse à évolution lente, certains patients présentent un risque élevé de progression de la maladie et d’issues plus graves telles que la cirrhose, le carcinome hépatocellulaire et la transplantation hépatique. Malgré les multiples études menées et les nombreux essais cliniques en cours, il n’existe pas de médicaments approuvés pour la NAFLD/NASH (*Non Alcoholic Steato-Hepatitis*), et le traitement doit donc se fonder sur des stratégies de modification du mode de vie. Cette revue explorera la définition et l’épidémiologie courantes de la NAFLD et de la NASH ainsi que les facteurs de risque et les conséquences de la maladie, tout en résumant les recommandations existantes pour le diagnostic, la stratification du risque et la prise en charge de la maladie.

## Abstract (de)

Die NAFLD (non-alcoholic fatty liver disease) ist die Lebermanifestation einer multisystemischen metabolischen Störung. Sie ist weltweit die Hauptursache für Lebererkrankungen, bei steigender Prävalenz. Obwohl es sich im Wesentlichen um eine stille Erkrankung mit langsamer Progression handelt, weisen manche Patienten ein hohes Risiko für ein Fortschreiten der Erkrankung und ernstere Entwicklungen wie z. B. Leberzirrhose, Leberzellkarzinom und Lebertransplantation auf. Trotz der diversen bereits erfolgten Studien und der zahlreichen aktuell durchgeführten klinischen Studien gibt es für die NAFLD/NASH (non-alcoholic steatohepatitis) keine zugelassenen Medikamente. Somit müssen die Behandlungsstrategien auf Änderungen des Lebensstils beruhen. Dieser Übersichtsartikel befasst sich mit der üblichen Definition und der Epidemiologie der NAFLD und der NASH sowie mit den Risikofaktoren und den Folgen der Erkrankung. Die verfügbaren Empfehlungen zur Diagnostik, Risikostratifizierung und Behandlung der Erkrankung werden zusammengefasst.

## Riassunto

La NAFLD (non-alcoholic fatty liver disease/steatosi epatica non alcolica) è la manifestazione epatica di un disturbo metabolico multisistemico. Nel mondo, è la causa principale di malattie epatiche nel mondo, con una prevalenza in aumento. Sebbene si tratti essenzialmente di una malattia silenziosa con una progressione lenta, alcuni pazienti presentano un rischio elevato di progressione della malattia con sviluppi più gravi come cirrosi, carcinoma epatocellulare e trapianto di fegato. Nonostante i vari studi già effettuati e gli studi clinici attualmente in corso, non esistono farmaci approvati per la NAFLD/NASH (NASH, nicht-alkoholischen Steatohepatitis/steatoepatite non alcolica). Pertanto, le strategie di trattamento devono basarsi su cambiamenti dello stile di vita. Questo articolo di revisione affronta la definizione comune e l’epidemiologia della NAFLD e della NASH, insieme ai fattori di rischio e alle conseguenze della malattia. Vengono riassunte le raccomandazioni disponibili per la diagnosi, la stratificazione del rischio e il trattamento della malattia.

## Introduction

Au cours des dernières décennies, la stéatose hépatique non alcoolique (*non-alcoholic fatty liver disease* en anglais, NAFLD) est devenue la maladie hépatique la plus fréquemment rencontrée dans le monde, touchant environ un quart de la population adulte [1]. L’épidémiologie mondiale de la NAFLD est directement liée à l’épidémie d’obésité et il est reconnu qu’elle est étroitement associée au syndrome métabolique et à ses composants individuels, tels que le diabète de type 2 (DT2), l’hyperlipidémie et l’hypertension [2]. En raison de l’augmentation rapide de la prévalence de ces comorbidités métaboliques, la NAFLD est en train de devenir une cause majeure de morbidité (cirrhose, cancer primaire du foie, transplantation du foie) et de mortalité liée aux maladies du foie [1]. Il est important de noter que le fardeau économique de la maladie va probablement augmenter au cours des prochaines années, et devrait dépasser la maladie du foie liée à l’alcool (*alcohol-related liver disease*, ALD) et devenir la principale indication de transplantation hépatique (TH) dans les pays occidentaux [3]. La prise en charge de cette maladie dans la pratique clinique présente quelques défis majeurs que nous allons aborder ici. L’établissement d’un système de classification distinct, la mise en place d’une stratégie de diagnostic et de surveillance efficace, l’identification des individus présentant un risque plus élevé de développer une maladie hépatique avancée et des complications et la mise en œuvre de politiques de prévention à l’échelle nationale en sont quelques-uns. Cette revue vise donc à explorer les tendances actuelles en matière d’épidémiologie, de diagnostic et de gestion de la NAFLD, tout en résumant les recommandations existantes pour la pratique clinique quotidienne.

## Définition de la NAFLD et de la NASH

La NAFLD est considérée comme la manifestation hépatique d’un syndrome métabolique multisystémique et la stéatohépatite non alcoolique (*non-alcoholic steatohepatitis*, NASH) est la composante inflammatoire de la NAFLD [4]. Selon la définition actuelle, la NAFLD est un diagnostic d’exclusion, défini par la présence d’une accumulation de stéatose dans plus de 5 % des hépatocytes en l’absence d’autres étiologies de maladies hépatiques (par exemple, hépatite virale, maladie hépatique auto-immune, etc.) ou de causes secondaires de stéatose hépatique (médicaments stéatogènes, consommation significative d’alcool défini comme ≥30 g par jour pour les hommes et ≥20 g par jour pour les femmes, etc.) [4, 5]. Sur le plan histologique, la NAFLD est un terme générique qui englobe un large spectre de maladies allant de la stéatose isolée (stéatose hépatique simple ou NAFL) à la NASH, cette dernière pouvant potentiellement conduire à une fibrose hépatique, une cirrhose ou un carcinome hépatocellulaire (CHC) [4, 5]. Au niveau cellulaire, outre la stéatose hépatique, la NASH est définie par une inflammation lobulaire et des signes de lésions hépatocytaires (caractérisés par une ballonisation des hépatocytes) avec différents degrés de fibrose [6].

### Nomenclature MAFLD contre NAFLD

En 2020, un groupe d’experts internationaux a réévalué la définition actuelle de la maladie stéatosique du foie et est parvenu à un consensus en faveur d’un changement de nomenclature, de la stéatose hépatique non alcoolique (NAFLD) à la stéatose hépatique associée à un dysfonctionnement métabolique (*metabolic (dysfunction)-associated fatty liver disease*, MAFLD) [7, 8]. En outre, ils ont établi un ensemble de critères diagnostiques “positifs” complets pour les patients adultes et pédiatriques [9]. Par conséquent, selon les nouveaux critères proposés, la MAFLD est définie par la présence d’une stéatose hépatique, en plus de l’un des trois critères suivants, à savoir le surpoids/l’obésité, la présence d’un DT2 ou la présence d’un dérèglement métabolique [7, 8].

Cette initiative est plus qu’un changement de nomenclature car elle a souligné la relation étroite entre les conditions métaboliques et la stéatose hépatique et elle a fait de la MAFLD un diagnostic d’inclusion qui n’exclut plus les patients atteints de maladies hépatiques concomitantes [8]. Bien que cet appel ait initialement suscité un débat dans le domaine, il a reçu un soutien important de la part des professionnels de la santé, des sociétés scientifiques, des infirmières ainsi que de représentants des patients, pharmaceutiques et réglementaires [10]. Il reste toutefois à déterminer si l’utilisation de la nouvelle nomenclature de MAFLD va intégrer la pratique clinique.

## Épidémiologie de la NAFLD et de la NASH

### Incidence et prévalence de la NAFLD et de la NASH

Il est impératif de comprendre l’incidence et la prévalence de la NAFLD et de la NASH, car elles sont directement liées à l’évolution des tendances en matière de TH et à l’augmentation des coûts des soins de santé associés. La NAFLD est sans aucun doute la principale étiologie des maladies chroniques du foie dans le monde, mais l’absence de critères de diagnostic cohérents rend sa véritable prévalence difficile à déterminer [2]. De plus, alors que la NAFLD peut être diagnostiquée par des modalités d’imagerie telles que l’échographie abdominale, le diagnostic de la NASH nécessite une histologie. Selon une méta-analyse récente, la prévalence mondiale de la NAFLD, lorsqu’elle est diagnostiquée par imagerie, est estimée à 32,4 % (intervalle de confiance [IC] 95 % 29,9 à 34,9) et elle a considérablement augmenté au fil du temps [1].

En raison de son association étroite avec un dérèglement métabolique, la NAFLD est plus fréquemment rencontrée chez les personnes présentant une composante du syndrome métabolique. Par exemple, la prévalence globale de la NAFLD et de la NASH chez les patients atteints de DT2 est estimée à 55,5 % (IC 95 %, 47,3 à 63,7) et 37,3 % (IC 95 %, 24,7-50,0 %), respectivement [11]. La NAFLD se retrouve également jusqu’à 80 % chez les patients obèses et chez plus de 90 % des patients subissant une chirurgie bariatrique [12].

L’incidence de la NAFLD dans la population générale est difficile à estimer et les données sont limitées. Une méta-analyse récente qui a utilisé le code CIM-10-CM pour la NAFLD a démontré une incidence allant de 28,0 pour 1000 personnes-années (IC 95 %, 19,3 à 40,6) en Israël à 50,9 pour 1000 personnes-années (IC 95 %, 44,8 à 57,4) en Asie, mais les taux sont probablement sous-estimés [1, 13]. Selon une étude de modélisation, la population NAFLD devrait augmenter de 30 % d’ici 2030, la Chine étant la plus touchée. Dans le même temps, la prévalence de la NASH devrait augmenter de 56 % et les maladies hépatiques avancées ainsi que la mortalité liée au foie devraient doubler [14].

La Suisse ne pouvait pas faire exception à la pandémie mondiale de NAFLD. Une étude de modélisation a estimé que d’ici 2030, il y aura 2.234.000 (1.918.000 à 2.553.000) cas de NAFLD, soit 24,3 % (20,9 à 27,8 %) de la population suisse totale. Alors que l’incidence de la maladie hépatique avancée est prévue d’augmenter de 40 % au cours de la même période [15].

### Conséquences de la NAFLD et de la NASH

L’histoire naturelle et les conséquences de la NAFLD ont fait l’objet de nombreuses études au cours des dernières décennies. Dans le passé, des études ont associé la NAFLD à une mortalité globale et liée au foie plus élevée que dans la population générale [16]. Cependant, la relation entre la NAFLD et la mortalité de toutes causes confondues reste débattue, certaines études ne rapportant aucune association [17].

Il est intéressant de noter que la NAFLD se caractérise par des degrés variables de progression de la maladie et de résultats cliniques, la majorité des patients présentant une maladie stable ou lentement progressive, tandis qu’une petite partie d’entre eux développe une fibrose avancée, une cirrhose et même un CHC [18]. Il est impératif de reconnaître les facteurs de risque de la progression de la maladie et des résultats indésirables pour élaborer des conseils efficaces en matière de soins aux patients.

Il a été démontré que le DT2 double le risque de fibrose avancée, de complications liées à la cirrhose et de mortalité liée au foie [19], tandis que la présence d’une obésité, d’une hyperlipidémie et d’une hypertension est également associée à un risque accru de maladie hépatique progressive [19]. L’âge avancé (> 60 ans), qui est lié à une plus longue durée de la maladie, joue également un rôle [20]. Il est de plus en plus évident que les patients atteints de NASH histologique, comparés à ceux atteints de NAFL ou de NAFLD, présentent un risque plus élevé de conséquences négatives telles que la progression de la maladie hépatique, la décompensation cirrhotique et le développement d’un CHC, et ont des taux de mortalité liés au foie plus élevés [21, 22]. Selon une méta-analyse d’études de biopsies hépatiques appariées de la NAFLD, la fibrose a progressé d’un stade en moyenne pendant 7,1 ans pour les patients NASH contre 14,3 ans pour les patients NAFLD, soit presque le double [21].

Il est important de noter que les patients atteints de NASH développent un CHC à un taux annuel 12 fois plus élevé que les patients atteints de NAFLD (5,77 contre 0,44 événements pour 1000 personnes-années) et ont un taux de mortalité annuel 1,7 fois plus élevé que les patients atteints de NAFLD (25,56 contre 15,44 événements pour 1000 personnes-années) [1]. Même les patients atteints de NASH non cirrhotique sont exposés à un risque accru d’hépato-carcinogenèse, qui peut être induit par la nécro-inflammation [23]. Malgré l’augmentation de la mortalité liée au foie chez les patients présentant à la fois un diagnostic de la NAFLD et de la NASH, les maladies cardiovasculaires semblent être la principale cause de décès, suivies par les tumeurs malignes extra-hépatiques (par exemple, le cancer colorectal et le cancer du sein) [5]. Enfin, les patients atteints de la NAFLD, en particulier ceux qui présentent une fibrose avancée, sont reconnus comme présentant un risque plus élevé de maladie grave due au SARS-Cov‑2 [24].

## Diagnostic et évaluation de la gravité de la maladie

### Diagnostic de la NAFLD

Dans la pratique quotidienne, la NAFLD est le plus souvent diagnostiquée par imagerie, bien qu’elle puisse également être identifiée histologiquement ou à partir de scores de risque cliniques. La modalité la plus couramment utilisée pour le diagnostic de la NAFLD est l’échographie abdominale, où la stéatose hépatique est caractérisée par une hyperéchogénicité hépatique et un flou de la vascularisation hépatique [25]. Une fibrose coexistante peut compliquer l’évaluation par échographie, car elle peut rendre l’écho-texture hépatique plus grossière. Une autre limite de l’échographie est sa faible sensibilité (<30 %) en cas de stéatose légère [26]. Les mesures basées sur l’imagerie par résonance magnétique (IRM) sont très sensibles pour l’évaluation de la stéatose hépatique (avec une sensibilité de 92 à 100 % et une spécificité de 92 à 97 %) et peuvent détecter une stéatose de 5 %. Par contre, l’IRM est réservée au cadre de la recherche, car elle est comparativement coûteuse et peu disponible dans la pratique courante. Aucune des modalités non invasives mentionnées ci-dessus ne permet de différencier la NAFLD de la NASH [27].

### Rôle de la biopsie du foie

La biopsie hépatique est la méthode actuellement acceptée pour différencier de manière fiable la NASH de la NAFLD et la méthode de référence pour l’évaluation de la fibrose hépatique [28, 29]. En outre, la biopsie hépatique est une exigence standard pour l’inscription aux essais cliniques pour les traitements de la NASH et de la NAFLD et la méthode la plus acceptée pour l’évaluation des progrès du traitement [30]. Cependant, étant donné que des modifications du mode de vie sont généralement recommandées pour tous les patients atteints de NAFLD et qu’aucun traitement spécifique de la NASH n’est actuellement approuvé, la nécessité d’une biopsie dans le cadre de la NAFLD reste controversée [30]. La biopsie hépatique présente certaines limites qui rendent sa mise en œuvre chez tous les patients atteints de NAFLD/NASH pour le diagnostic et l’évaluation de la sévérité et de la progression de la maladie impossible. Bien qu’elle soit généralement bien tolérée, il s’agit d’une procédure invasive qui comporte un risque de complications telles que des saignements, des infections, des fuites biliaires, des lésions d’autres organes et un risque de mortalité rare [31]. Il semble également exister une certaine variation dans l’échantillonnage et l’interprétation par les observateurs qui peut affecter l’intégrité du diagnostic [32].

### Tests non invasifs pour l’évaluation de la gravité de la maladie

Au cours des dernières décennies, les limites de la biopsie hépatique, combinées à l’épidémie croissante de NAFLD, ont stimulé le développement de stratégies alternatives non invasives afin de servir d’outils de diagnostic et de pronostic. Les stratégies non invasives s’appuient soit sur des scores et des biomarqueurs sériques, soit sur des mesures de l’élasticité du foie par imagerie, à l’aide de techniques basées sur l’échographie ou l’IRM.

#### Scores sériques et biomarqueurs non invasifs

Les concentrations d’enzymes hépatiques ont traditionnellement été utilisées par les cliniciens pour évaluer les patients atteints de maladies du foie. Cependant, les enzymes hépatiques sont souvent normales chez ces patients et peuvent ne pas refléter la gravité histologique [33]. Le degré de fibrose étant le facteur prédictif le plus fort de la morbidité et de la mortalité liées aux maladies du foie, de nombreux efforts de recherche ont été déployés pour mettre au point des scores sériques simples et non invasifs permettant d’estimer le degré de fibrose d’un patient [22].

**Figure 1:**
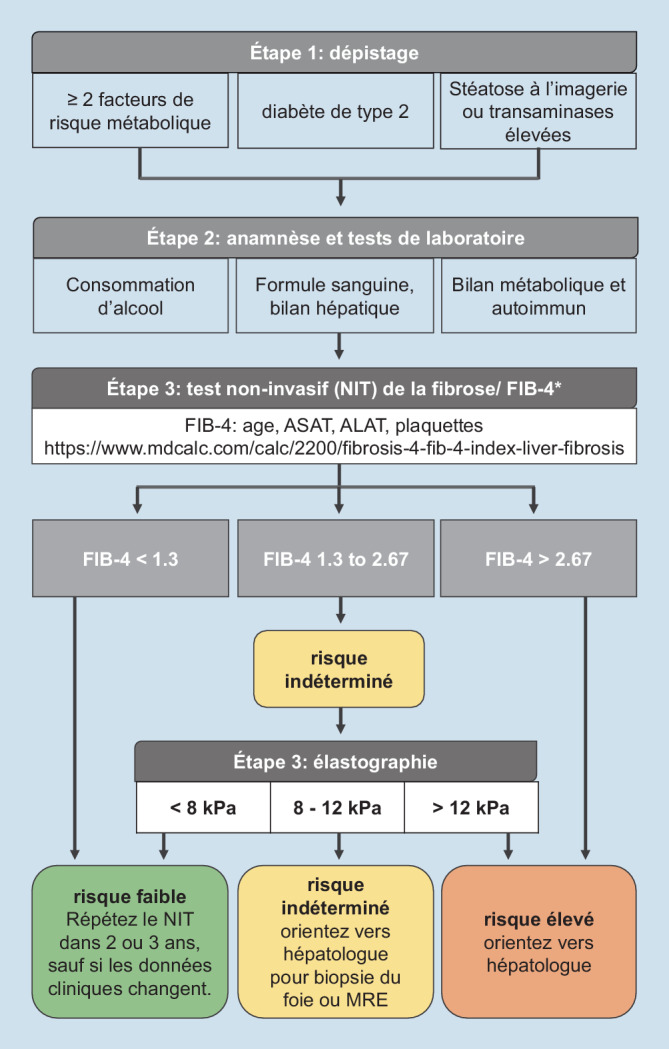

Les plus couramment utilisés sont le score de fibrose de la NAFLD (NFS), l’indice de fibrose FIB‑4 et l’indice du rapport entre l’aspartate aminotransférase et les plaquettes (APRI), qui sont calculés à partir de paramètres de laboratoire, cliniques et démographiques couramment disponibles ([34]; Tab. 1). Bien que ces scores aient une précision modérée, leur valeur prédictive négative élevée en font des outils utiles pour exclure une fibrose avancée [35, 36]. Le FIB‑4 a été particulièrement proposé dans le cadre d’un algorithme de soins aux patients en tant qu’outil de dépistage afin de sélectionner les patients présentant un risque plus élevé de fibrose avancée qui pourraient nécessiter une évaluation spécialisée avec une mesure de l’élasticité du foie ou éventuellement une biopsie du foie ([37]; Fig. 1). Le FIB‑4 a également été identifié comme un prédicteur indépendant de la mortalité et des résultats liés au foie dans la NAFLD [38].

**Table 1:** 

Test non invasif de la fibrose	Paramètres	Algorithme de calcul
*Tests indirects de la fibrose*		
FIB‑4	Age, ASAT, ALAT, plaquettes	Age (years) x ASAT (U/L) / [plaquettes (10^9^/L) x ALAT^1/2^ (U/L)]
NFS	Age, BMI, diabète, ASAT, ALAT, plaquettes, albumine	−1,675 + 0,037 x age (years) + 0,094 x BMI (kg/m^2^) + 1,13 x diabète (oui = 1, non = 0) + 0,99 x ASAT/ALAT − 0,013 x plaquettes (10^9^/L) − 0,66 x albumine (g/dL)
APRI	ASAT, ALAT, plaquettes	[ASAT/limite supérieure de l’intervalle normal de l’ASAT] x 100 / plaquettes (10^9^/L)
*Tests directs de la fibrose*		
ELF	HA, PIIINP, TIMP‑1	2,494 + 0,846 In(C HA) + 0,735 In(C PIIINP) + 0,391 In(C TIPM-1)

*ASAT* aspartate transaminase, *ALAT* alanine transaminase, *BMI* body mass index, *HA* hyaluronic acid, *PIIINP* procollagen III amino terminal peptide, *TIMP‑1* tissue inhibitor of metalloproteinase 1

Parmi les divers biomarqueurs sanguins directs de la fibrose, le score ELF (*Enhanced Liver Fibrosis*) a été le plus étudié et son utilisation est recommandée par le UK *National Institute for Health and Care Excellence *avant l’orientation vers un spécialiste en hépatologie [39, 40]. Cependant, selon une récente méta-analyse, le test a montré une performance diagnostique limitée dans des contextes de faible prévalence [41].

#### Imagerie

L’évaluation de la fibrose par imagerie non invasive repose principalement sur les techniques d’élastographie qui mesurent l’élasticité du foie en quantifiant la vitesse de propagation dans le parenchyme hépatique d’une onde mécanique. Les tissus fibrotiques plus rigides propagent les ondes plus rapidement, ce qui entraîne des valeurs plus élevées (mesurées en kilopascals, kPa) [42]. Les techniques d’élastographie peuvent être basées sur l’échographie, comme l’élastographie impulsionnelle à vibration contrôlée (VCTE) ou FibroScan, l’élastographie point Shear Wave ou l’élastographie ultrasonore impulsionnelle 2D en mode Shear Wave Elastography, ou sur l’IRM, comme l’élastographie par résonance magnétique [43].

En ce qui concerne le VCTE/Fibroscan, afin d’exclure une fibrose significative (stade de fibrose F2–F3), une faible valeur seuil de 8,0 kPa est recommandée selon le guide des soins cliniques de l’Association Américaine de Gastroentérologie (AGA) [37]. En effet, une grande étude européenne qui a recruté 1073 patients atteints de NAFLD dans 10 centres hépatiques a démontré qu’une faible valeur seuil de 8.0 kPa a une sensibilité de 93% pour l’exclusion de la fibrose avancée (stade de fibrose ≥F3), ce qui a également été soutenu par une revue systématique récente [35, 44]. Il a été constaté qu’une valeur de 12,1 kPa sur le VCTE a une valeur prédictive positive de 88% pour le diagnostic d’une fibrose significative chez les patients d’une clinique d’hépatologie [41]; c’est pourquoi une valeur arrondie de 12,0 kPa est recommandée comme seuil supérieur [37]. Les mesures de l’élasticité du foie sont également corrélées au risque de CHC et aux complications de la cirrhose [45], tandis que les critères de Baveno VI combinent la VCTE et la valeur des thrombocytes dans le sang pour identifier les patients à risque de varices œsophagiennes qui doivent être traités [46].

## Prise en charge de la NAFLD et de la NASH

La NAFLD et la NASH sont des affections multifactorielles avec des dérèglements métaboliques coexistants variables. Par conséquent, la prise en charge initiale, que ce soit dans le cadre de soins primaires ou secondaires, doit commencer par la recherche d’autres étiologies possibles de la maladie hépatique et de la consommation concomitante d’alcool, mais aussi par le contrôle des éventuelles comorbidités métaboliques comme le DT2 et, surtout, par le calcul du risque d’événements cardiovasculaires. Pour les raisons susmentionnées, la prise en charge de la NAFLD/NASH est difficile à standardiser et requiert une approche plus personnalisée et holistique qui fait appel à des cliniciens de différentes spécialités, tels que des gastroentérologues, des hépatologues, des endocrinologues, des chirurgiens viscéraux, des spécialistes de la médecine interne et des médecins généralistes. Le Tab. 2 présente une comparaison de toutes les recommandations actuelles des associations de gastroentérologie et d’hépatologie en Europe, aux États-Unis et en Asie (Tab. 2).

**Table 2:** 

	EASL, 2016 [4]	AASLD, 2018 [5]	APASL, 2020 [81]
Dépistage	1. Les personnes présentant des enzymes hépatiques anormales de manière persistante. 2. Les patients présentant une résistance à l’insuline et/ou une obésité ou un MetS	1. Le dépistage de routine de la NAFLD dans les groupes à haut risque n’est pas conseillé	1. Le dépistage du MAFLD par échographie doit être envisagé dans les patients en surpoids/obésité, DT2 et MetS
Evaluation de la fibrose	1. NFS et FIB-4 peuvent être utilisés pour la stratification du risque afin d’exclure une maladie grave. 2. Les patients présentant un risque moyen à élevé doivent être orientés vers un hépatologue afin de subir une élastographie et/ou biopsie du foie. 3. L’identification d’une fibrose avancée ou d’une cirrhose par des outils non invasifs est moins précise et doit être confirmée par une biopsie du foie. 4. La NASH doit être diagnostiquée par une biopsie du foie	1. NFS, FIB-4 ou VCTE ou MRE pour identifier les personnes présentant un risque de fibrose avancée (F3 ou F4). 2. Les patients atteints du MetS sont à risque de développer une NASH et doivent être ciblés pour une biopsie du foie	1. VCTE ou SWE et les biomarqueurs sanguins et scores de fibrose ou des combinaisons peuvent exclure le risque élevé de fibrose significative ou avancée (F2–F4). 2. La confirmation d’une fibrose significative ou avancée par des outils non invasifs est moins précise et doit être confirmée par une biopsie du foie. 3. La biopsie du foie est le test de choix pour la NASH
Intervention sur le mode de vie	1. Objectif de perte de poids totale de 7 à 10 %. 2. Défaut énergétique de 500–1000 kcal/jour, pour une perte de poids de 500–1000 g/semaine. 3. Consommer l’alcool en dessous du seuil de risque et éviter les boissons et aliments contenant du fructose. 4. 150–200 min/semaine d’activité physique aérobique d’intensité modérée en 3–5 séances	1. Objectif de perte de poids de 7 à 10 %. 2. Combinaison d’un régime hypocalorique (réduction de 500 à 1000 kcal/jour) et d’un programme d’activité physique d’intensité modérée. 3. Pas de consommation de grandes quantités d’alcool	1. Objectif de perte de poids de 7 à 10 %. 2. Restriction énergétique et exclusion des aliments industriels et riches en fructose. Le régime méditerranéen est conseillé. 3. La combinaison du régime alimentaire et de l’exercice physique est plus efficace. 4. Exercice aérobique ou entraînement en résistance, selon la condition physique
Pharmacothérapie	1. Réservée aux patients atteints de la NASH, en particulier à ceux qui présentent une fibrose significative (≥ F2) ou avec une maladie moins grave, mais a risque élevé de progression (diabète, MetS, augmentation persistante de l’ALT, nécro-inflammation). 2. La pioglitazone (hors indication en dehors de DT2) et la vitamine E ou une combinaison peut être utilisée dans la NASH. 3. Les statines peuvent être utilisées pour prévenir le risque cardiovasculaire	1. Réservée aux personnes atteintes de NASH avec une fibrose prouvée par biopsie. 2. La pioglitazone peut être utilisée chez les patients avec ou sans DT2 avec une NASH prouvée par biopsie. 3. La vitamine E (800 UI/jour) peut être utilisée chez les patients sans DT2 dont la NASH a été prouvée par biopsie. 4. Les statines peuvent être utilisées pour traiter la dyslipidémie chez les patients atteints de la NAFLD et de la NASH	1. Aucune recommandation spécifique de pharmacothérapie pour la MAFLD. 2. Les statines doivent être envisagées chez tous les patients atteints de MAFLD présentant une hyperlipidémie

*AASLD* Association Américaine pour l’Étude du Foie, *APASL* Association Asie-Pacifique pour l’Étude du Foie, *EASL* Association Européenne pour l’Étude du Foie, *MetS* syndrome métabolique

### Investigation et gestion de la consommation d’alcool

Au niveau mondial, la consommation d’alcool est associée à une augmentation de pathologies multiples [47]. Dans le domaine de l’hépatologie, l’impact de l’alcool chez les patients atteints de NAFLD reste un sujet de débat. Selon le récent consensus suggérant un changement de nomenclature de la NAFLD à la MAFLD, l’alcool ne doit plus être exclu pour le diagnostic de MAFLD [7, 8]. Cependant, l’évaluation du niveau de consommation d’alcool dans la pratique clinique quotidienne est un défi car il n’existe ni de questionnaire standard largement reconnu ni de biomarqueurs sériques précis [48]. Actuellement, selon les directives de l’Association Européenne pour l’Étude du Foie (EASL), la consommation excessive d’alcool est définie comme une consommation régulière de > 20 g pour les femmes et > 30 g pour les hommes par jour [48]. Des études récentes ont montré que la consommation d’alcool dans les limites actuellement acceptées chez les patients atteints de NAFLD peut être nuisible, tandis que d’autres études ont conclu à un effet protecteur [49, 50]. Une vaste étude coréenne qui a recruté des patients atteints de NAFLD avec de faibles scores sériques non invasifs de fibrose (NFS et FIB-4) a suggéré qu’une consommation légère (1–9,9 g/jour) ou modérée (10–29,9 g/jour pour les hommes ou 10–19,9 g/jour pour les femmes) était indépendamment associée à la progression de la fibrose [49]. Une autre étude récente qui a évalué l’alcool à l’aide d’un biomarqueur sérique a révélé qu’une consommation modérée d’alcool est associée à une fibrose avancée et que les patients atteints de DT2 sont plus à risque [51]. Compte tenu des preuves croissantes de l’effet négatif d’une consommation modérée d’alcool dans la progression de la fibrose, la consommation d’alcool devrait être découragée chez ces patients [4]. Chez les sujets avec fibrose avancée, voire une cirrhose, la consommation d’alcool doit être proscrite afin d’éviter la progression de l’hépatopathie ou développement de complications.

### Recherche et gestion des comorbidités

Même avant le consensus sur la définition de la MAFLD, le diagnostic et la gestion des comorbidités métaboliques faisaient partie de la pratique quotidienne dans le domaine de la NAFLD. Dans un premier temps, le diagnostic d’une autre hépatopathie coexistente doit être effectué, tel qu’une hépatite virale, une hépatopathie médicamenteuse, une hémochromatose, une maladie auto-immune et des étiologies moins courantes comme la maladie de Wilson [52]. En ce qui concerne l’hépatite virale et la NAFLD, des études ont montré que la coexistence des deux est associée à une hépatopathie plus avancée. Par exemple, les patients atteints d’hépatite C et de stéatose présentent un risque plus élevé d’évènements plus graves, tels que des événements cardiovasculaires [53], et les patients atteints d’hépatite B et de syndrome métabolique traités par des analogues nucléosidiques présentent un risque plus élevé de résistance virale, de développement de CHC et de progression de la maladie [54]. Par conséquent, un dépistage et une prise en charge appropriés doivent être effectués.

### Gestion des maladies cardiovasculaires

Chez les patients atteints de NAFLD, un dépistage systématique des maladies cardiovasculaires devrait être mis en place, car les événements cardiovasculaires augmentent la morbidité et la mortalité. L’une des cibles du traitement doit être la dyslipidémie. Le traitement par statine s’est avéré sûr pour les patients atteints de maladies hépatiques, même ceux qui présentent des taux élevés de transaminases, et doit être prescrit conformément aux directives [55, 56]. Par conséquent, l’utilisation de statines et/ou l’ézétimibe devrait être administrée chez les patients NAFLD présentant un risque cardiovasculaire élevé [57].

### Mesures hygiéno-diététiques

Le mode de vie représente le facteur le plus pertinent pour la NAFLD, en tant que composante hépatique du syndrome métabolique. Bien que de nombreuses données aient démontré l’effet bénéfique de la modification du mode de vie dans la NAFLD, la complexité du sujet rend difficile la formulation de recommandations cliniques fondées sur des preuves [58].

#### Régime alimentaire

On sait que la composition en macronutriments de l’alimentation influe sur le dépôt de graisse hépatique, mais aucun régime spécifique en macronutriments ne s’est avéré bénéfique pour la NASH (régime riche en protéines, riche en glucides/faible en graisses, faible en glucides/riche en graisses etc.) [58].

Une étude qui visait à étudier l’effet d’un régime riche en protéines sur le contenu lipidique intrahépatique a montré que les sujets ayant suivi un régime riche en protéines ont obtenu une diminution plus importante du contenu lipidique intrahépatique (43 %) par rapport à ceux ayant suivi un régime normal ou faible en protéines (37 % et aucune réduction, respectivement), mais les différences ont été attribuées à la différence de concentration en glucides dans chaque régime [59]. Un régime riche en glucides et pauvre en graisses peut être bénéfique à moyen terme s’il entraîne un déficit calorique global [60], mais la qualité des graisses doit toujours être prise en compte [61]. En outre, on s’inquiète de plus en plus des effets indésirables du suivi à long terme d’un régime riche en glucides [62].

Il a notamment été démontré que le régime méditerranéen a des effets bénéfiques sur la NAFLD. De récents essais de contrôle randomisés (ECR) ont montré que l’adhésion à un régime méditerranéen conduit indépendamment à une diminution de la quantité de la stéatose intrahépatique [63–65], tandis qu’une grande étude prospective observationnelle a rapporté une association inverse entre le régime méditerranéen et la NAFLD [66]. En outre, l’exclusion des facteurs favorisant la NAFLD, comme les aliments industriels et les aliments et boissons à haute concentration de fructose ajouté, est recommandé [4].

D’autre part, la perte de poids par simple restriction calorique est considérée comme la stratégie la plus appropriée et la plus efficace pour les patients atteints de NAFLD [5]. Un régime hypocalorique, quelle que soit sa composition, peut entraîner une diminution du poids corporel, des taux de transaminases, de la graisse corporelle totale et de la graisse viscérale [67]. Une étude récente a montré que le degré de perte de poids après un régime hypocalorique et le degré de régression histologique de la NAFLD sont fortement associés [60] et cette corrélation a été récemment confirmée par une méta-analyse [68]. Des études ont montré qu’une perte de poids d’au moins 5 % est nécessaire pour l’amélioration de la stéatose hépatique dans la NASH [67, 69], tandis qu’une perte de poids de plus de 10 % peut conduire à une résolution complète de la NASH dans 90 % des cas et à une régression de la fibrose dans 45 % des cas [60]. Cependant, le pourcentage de patients capables d’atteindre cet objectif est faible, il faut donc mettre l’accent sur l’objectif d’une perte de poids de 7 à 10 %, en utilisant une approche personnalisée, tenant compte de la cuisine, du coût et des habitudes locales [60].

#### Activité physique

Il a été démontré que l’activité physique réduit la proportion de graisse intrahépatique et les marqueurs de lésions hépatocellulaires, indépendamment de la perte de poids [70]. Cependant, lorsque l’activité physique est associée à un régime alimentaire, les bénéfices sont considérablement accrus [70]. Bien que les études évaluant l’effet de l’exercice sur l’histologie dans la NASH manquent, une méta-analyse a montré que l’exercice améliore les scores histologiques mais pas les niveaux de l’alanine transaminase (ALAT) [71]. La durée et le type d’exercice optimaux restent également indéterminés. Une vaste étude coréenne a démontré que l’exercice modéré cinq fois par semaine est bénéfique pour la prévention ou l’amélioration de la NAFLD, indépendamment du poids corporel [52]. Selon les directives de pratique clinique de l’EASL, de l’Association Européenne pour l’Étude du Diabète et de l’Obésité et les directives de l’Association Américaine pour l’Étude du Foie, il est recommandé aux patients atteints de la NAFLD de pratiquer une activité physique aérobie d’intensité modérée pendant 150 à 200 min par semaine, en trois à cinq séances [4, 72]. Dans l’ensemble, tous les patients atteints de la NAFLD, indépendamment de leur poids et de leur adiposité, devraient recevoir une éducation sur la nutrition, l’activité physique et la restriction d’alcool [4].

### Pharmacothérapie

L’EASL propose de traiter les patients présentant une fibrose significative ou une maladie moins sévère mais présentant un risque élevé de progression de la maladie (par exemple, avec un DT2, un syndrome métabolique, une augmentation persistante de l’ALAT, une nécro-inflammation élevée à l’histologie) [4]. À l’heure actuelle, aucun médicament n’est approuvé pour la NASH en particulier, avec seulement quelques médicaments entrant dans la phase III des essais cliniques. Par conséquent, tout traitement médicamenteux recommandé serait hors indication ou dans le cadre d’essais cliniques. La pathophysiologie complexe de la maladie se traduit par une pléthore de cibles thérapeutiques potentielles, comprenant certains médicaments ciblant l’homéostasie métabolique, comme le lanifibranor, d’autres agissant directement sur le foie, comme le resmetirom et l’acide obéticholique et les analogues du récepteur du glucagon-like peptide‑1 (GLP-1) comme le semaglutide [73].

Lanifibranor est un agoniste des récepteurs activés par les proliférateurs de peroxysomes (PPAR) qui a terminé les essais de phase II. Agissant sur de multiples mécanismes métaboliques dont l’adipogenèse, l’inflammation et la fibrose, lanifibranor a atteint son critère principal de diminution du score histologique stéatose – activité – fibrose de > 2 points sans aggraver la fibrose chez 49 % des patients [74].

Les résultats de l’étude de phase II du resmetirom sont encourageants, montrant une réduction de la stéatose hépatique de 30 % à 12 semaines et une résolution significative de la NASH, tandis que la phase III présente déjà des résultats positifs en termes de stéatose et de rigidité du foie [75, 76]. D’autre part, une grande étude de phase III sur l’acide obéticholique a montré une amélioration d’au moins un stade de fibrose mais pas de résolution de la NASH [77].

Les agonistes des récepteurs du GLP‑1 ont de multiples modes d’action et sont connus pour induire une perte de poids significative et une amélioration des paramètres cardiométaboliques [78]. L’essai de phase II du semaglutide a montré une résolution de la NASH sans aggravation de la fibrose chez 56 % des patients par rapport à 20 % pour le placebo, sans toutefois réussir à améliorer la fibrose [79].

Finalement, selon un grand ECR, la vitamine E, qui a un mode d’action antioxydant important, peut améliorer la stéatose, l’inflammation et le ballonisation et a résolu la NASH dans 36 % des cas, contre 21 % dans le bras placebo [52]. Une méta-analyse de 5 ECR a démontré que la vitamine E améliore les transaminases hépatiques chez les patients adultes atteints de NAFLD sans DT2 [80].

Enfin, il est probable que dans un avenir proche, ces médicaments joueront un rôle important dans la prise en charge des patients atteints de la NAFLD/NASH avec ou sans DT2, mais des études prospectives et des ECR supplémentaires sont nécessaires.

## Message à retenir

La NAFLD est la composante hépatique d’un trouble métabolique multi systémique qui est la principale cause de maladie du foie dans le monde, avec une prévalence qui ne cesse d’augmenter. Bien qu’il s’agisse principalement d’une maladie silencieuse et à progression lente, certains patients présentent un risque élevé de progression de la maladie et de résultats plus graves tels que la cirrhose, le carcinome hépatocellulaire et la transplantation hépatique. En étudiant et en comprenant mieux l’histoire naturelle et les facteurs de risque, il est impératif de développer des parcours de soins cliniques efficaces pour le diagnostic et la stratification du risque pour les patients. Bien que la biopsie hépatique reste la méthode de référence pour l’évaluation de la fibrose, il convient d’accorder une plus grande attention aux stratégies non invasives. Enfin, la prise en charge de la NAFLD et de la NASH nécessite la collaboration d’une équipe multidisciplinaire qui doit mettre l’accent sur l’identification d’autres maladies hépatiques possibles et de comorbidités métaboliques et cardiovasculaires susceptibles de modifier sensiblement le pronostic. Actuellement, il n’existe aucun médicament approuvé pour la NAFLD; le traitement doit donc se concentrer sur des stratégies de modification du mode de vie.

## Abréviations

AASLD: Association Américaine pour l’Étude du Foie
ALD: Maladie du foie liée à l’alcool (*alcohol-related liver disease*)
APRI: Indice du rapport entre l’aspartate aminotransférase et les plaquettes
CHC: Carcinome hépatocellulaire
DT2: Diabète de type 2
EASL: Association Européenne pour l’Etude du Foie
ELF: Enhanced Liver Fibrosis
ECR: Essai contrôlé randomisé
GLP‑1: Glucagon-like peptide‑1
IC: Intervalle de confiance
IRM: Imagerie par résonance magnétique
kPa: Kilopascals
MAFLD: Maladie du foie gras associée à une dysfonction métabolique (*Metabolic Associated Fatty Liver Disease*)
MRE: Élastographie par résonance magnétique
NAFL: Stéatose hépatique simple
NAFLD: Stéatose hépatique non alcoolique
NASH: Stéatohépatite non alcoolique
NFS: NAFLD fibrosis score
SGLT2: Sodium-glucose co-transporteur‑2
TH: Transplantation hépatique
US: Échographie
VCTE: Élastographie impulsionnelle à vibration contrôlée

## Conformité aux directives éthiques

### Conflit d’intérêts

N.M. Tagkou et N. Goossens déclarent ne pas avoir de conflit d’intérêt.

Pour cet article, les auteurs n’ont réalisé aucune étude sur les humains ou les animaux. Les études réalisées étaient conformes aux normes éthiques indiquées dans chaque cas.
